# Methylxanthine Treatment in Neonates Admitted to the Special Care Unit: An Observational Study in Low-Resource Settings

**DOI:** 10.3390/children13010054

**Published:** 2025-12-30

**Authors:** Martina Borellini, Francesco Cavallin, Nasri Mfaume, Eleni Hagos Giday, Tarikua Endrias, Jiksa Tolera, Paolo Belardi, Fabio Manenti, Giovanni Putoto, Luigi Pisani, Daniele Trevisanuto

**Affiliations:** 1 Doctors with Africa CUAMM, Iringa P.O. Box 11, Tanzania; m.borellini@cuamm.org (M.B.); p.belardi@cuamm.org (P.B.); 2 Independent Statistician, 36020 Solagna, Italy; cescocava@libero.it; 3 Tosamaganga Regional Referral Hospital, Iringa P.O. Box 11, Tanzania; nasrimfaume4@gmail.com; 4 Doctors with Africa CUAMM, Wolisso P.O. Box 250, Ethiopia; e.hagos@cuamm.org; 5 St. Luke Catholic Hospital and College of Nursing and Midwifery, Wollisso P.O. Box 250, Ethiopia; tarikuaendrias1@gmail.com (T.E.); jiksatolera@gmail.com (J.T.); 6 Doctors with Africa CUAMM, 35121 Padua, Italy; f.manenti@cuamm.org (F.M.); g.putoto@cuamm.org (G.P.); 7 Department of Precision-Regenerative Medicine and Jonic Area (DiMePRe-J), Section of Anesthesiology and Intensive Care Medicine, University of Bari “Aldo Moro”, 70121 Bari, Italy; luigipisani@gmail.com; 8 Mahidol Oxford Tropical Research Unit (MORU), Bangkok 10400, Thailand; 9 Department of Woman’s and Child’s Health, University of Padua, 35128 Padova, Italy

**Keywords:** apnea of prematurity, caffeine, methylxanthine, low-resource setting, prematurity

## Abstract

**Highlights:**

**What are the main findings?**

- In two Sub-Saharan settings, methylxanthine treatment for apnea of prematurity was limited to aminophylline, which was given to around 12% of infants admitted to the special care units.
- Overall, the treatment was appropriately given to most eligible neonates, although a considerable subgroup of very preterm infants did not receive aminophylline prophylaxis.

**What is the implication of the main finding?**

- Further studies may investigate the reasons for protocol incompliance regarding aminophylline treatment and healthcare staff’s opinions on such an aspect.
- Use of caffeine in these settings may improve adherence to therapeutic protocols.

**Abstract:**

**Background**: The appropriate identification of target patients for methylxanthine therapy may optimize resource allocation and improve clinical outcomes, but data on routine care in low-resource settings are limited. Our study assessed methylxanthine use in clinical practice in two Sub-Saharan settings. **Methods**: This retrospective, registry-based study investigated methylxanthine use in newborns who were admitted to Tosamaganga Hospital (Tanzania) and Wolisso Hospital (Ethiopia) in 2022–2023. The prevalence and type of methylxanthine treatment were investigated. Neonates receiving methylxanthine were compared to those not receiving it in terms of baseline characteristics, clinical data, treatments, and discharge information. All data were retrieved from local registries. **Results**: Aminophylline was administered to 196/1674 neonates (11.7%), while caffeine was not available in these settings. This treatment was more common in preterm and smaller infants (*p* < 0.0001), asphyxiated neonates (*p* < 0.0001), and the sickest patients (*p* < 0.001). The need for respiratory support (*p* < 0.0001), intravenous lines (*p* < 0.0001), and antibiotic therapy (*p* < 0.0001), as well as the length of hospital stay (*p* < 0.0001) and mortality rate (*p* < 0.0001), were higher in neonates receiving aminophylline. **Conclusions**: In two Sub-Saharan settings, methylxanthine treatment was limited to aminophylline, which was given to around 12% of infants admitted to the special care units. Overall, the treatment was appropriately given to most eligible neonates, although a considerable subgroup of very preterm infants did not receive aminophylline prophylaxis. Further studies may investigate the reasons for protocol incompliance regarding aminophylline treatment and healthcare staff’s opinions on such an aspect.

## 1. Introduction

Prematurity is the main cause of under-five mortality and long-term morbidity [1]. In 2020, over 13 million newborn babies were estimated to be born before 37 weeks gestation worldwide, without relevant changes over the last decade [2].

The healthcare burden on morbidity is expected to rise because of the increasing survival of very preterm infants. Hence, the prevention of prematurity-related complications remains a global health urgency [3,4].

Respiratory distress and apnea of prematurity are common complications in preterm infants [5]. These have been mainly treated with invasive ventilatory support [5,6], but non-invasive respiratory strategies have been implemented in recent decades to reduce the consequences of mechanical ventilation [6,7]. Nonetheless, these strategies may be insufficient in patients with severe respiratory distress or frequent episodes of apnea [7].

In such patients, methylxanthines such as caffeine and aminophylline are routinely given to prevent conversion from non-invasive to invasive ventilatory support [5]. The drug effects include improved stimulation of respiratory drive and enhanced diaphragmatic contractility, with clinical advantages in reducing mechanical ventilation, apnea and bronchopulmonary dysplasia, and long-term neurodevelopmental impairment [8]. Caffeine is the most used methylxanthine thanks to its ease of use, long half-life, and extensive therapeutic range and is included in the list of essential drugs by the World Health Organization [9,10]. Although caffeine is recommended as first-line therapy (followed by aminophylline when caffeine is unavailable), aminophylline is still widely used in low-resource settings due to cost and shortage of caffeine [11,12].

Appropriate identification of target patients for methylxanthine treatment may optimize resource allocation and improve the outcome of preterm infants. However, data on methylxanthine use in clinical practice in low-resource settings are limited. Our study assessed this aspect in two Sub-Saharan settings, aiming to provide useful information to clinicians.

## 2. Materials and Methods

### 2.1. Study Design

This retrospective, observational, registry-based study investigated methylxanthine use in newborns who were admitted to Tosamaganga Hospital (Tanzania) and Wolisso Hospital (Ethiopia) in 2022–2023. The study was approved by the local Review Boards (protocol number NIMR/HQ/R.8a/Vol.IX/5055 and Ref. No BFO/HQ/093/17). The local registries are parts of the Critical Care Asia Africa (CCAA) federated network of registries. The registries received ethical approval, and a waiver of informed consent was provided from the local review boards [13,14]. The investigations were conducted in accordance with the principles of the Declaration of Helsinki.

### 2.2. Setting

Tosamaganga Regional Referral Hospital (Tosamaganga Hospital) is a non-profit, secondary-level hospital in the Iringa District Council; this is a rural district in Southwestern Tanzania covering around 320,000 people. St. Luke Catholic Hospital in Wolisso (Wolisso Hospital) is a non-profit, tertiary-level hospital in Wolisso town; this is the capital of the Southwest Shoa Zone in the Oromiya region (Ethiopia) with about 1.1 million inhabitants. Both hospitals account for about 3500–3600 deliveries per year. Aminophylline is the only available methylxanthine for the prevention and treatment of apnea of prematurity. At Tosamaganga Hospital, all newborns with birth weight < 1600 g or gestational age < 34 weeks receive prophylactic treatment with aminophylline; the treatment is stopped before discharge in patients reaching 1500 g or 34 weeks gestation. At Wolisso Hospital, all newborns with birth weight < 1500 g or gestational age < 34 weeks receive prophylactic treatment with aminophylline; the treatment is stopped before discharge in patients without apnea episodes in the last 7 days or reaching 34 weeks of gestation. In both hospitals, all newborns with apnea episodes may receive aminophylline according to the decision of the attending physician.

### 2.3. Patients

All neonates admitted to the Special Care Units of the participating hospitals between 1 January 2022 and 31 December 2023 were retrospectively included in the study. The only exclusion criterion was the unavailability of information on methylxanthine use.

### 2.4. Data Collection

All data were retrieved from the local registries and collected in a dedicated electronic spreadsheet after being anonymized. Data collection included the participating hospital, baseline characteristics (source of admission, maternal history, delivery, neonatal demographics), clinical information (Apgar score at 5 min, temperature, oxygen saturation), respiratory support, aminophylline treatment, neonatal diagnoses, and discharge information (hospital length of stay, respiratory support during hospital stay, intravenous line, antibiotic therapy, kangaroo mother care, feeding, and discharge status).

### 2.5. Statistical Analysis

Numerical data were summarized as median and interquartile range (IQR), while categorical data were summarized as absolute and relative frequency (percentage). Data were compared between the two groups using the Mann–Whitney test (continuous data) and Fisher’s test or the chi-squared test (categorical data). Effect sizes were reported as median difference or odds ratio (ORs) with 95% confidence intervals (CIs). All tests were two-sided, and a *p*-value <0.05 was considered statistically significant. Statistical analysis was carried out using R 4.4 (R Foundation for Statistical Computing, Vienna, Austria) [15].

## 3. Results

During the study period, aminophylline was administered to 196 out of 1674 neonates (prevalence of 11.7%; 95% confidence interval 10.2–13.4%) who were admitted to the special care units of the participating hospitals. Information on methylxanthine use was unavailable in 280 other neonates.

The baseline characteristics are summarized in Table 1. Receiving aminophylline was more frequent in outborn neonates (OR 2.29, 95% CI 1.51 to 3.48; *p* < 0.0001), multiple births (OR 4.41, 95% CI 3.03 to 6.41; *p* < 0.0001), and neonates born through assisted breech (OR 3.97, 95% CI 1.95 to 9.07; *p* < 0.0001). Mothers with preeclampsia/eclampsia were more likely to give birth to a neonate requiring aminophylline (OR 3.65, 95% CI 1.85 to 8.09; *p* < 0.0001). Meconium-stained amniotic fluid was associated with a lower likelihood of receiving aminophylline (OR 0.55, 95% CI 0.33 to 0.90; *p* = 0.002). Overall, aminophylline was administered more frequently in preterm infants (OR 17.48, 95% CI 11.77 to 25.97; *p* < 0.0001), low-birth-weight infants (OR 20.16, 95% CI 12.95 to 31.40; *p* < 0.0001), and those with an Apgar score < 7 at 5 min (OR 2.37, 95% CI 1.66 to 3.41; *p* < 0.0001), lower temperature (median difference −1.0, 95% CI −1.2 to −0.8; *p* < 0.0001), or lower transcutaneous oxygen saturation (median difference −1.0, 95% CI −1.9 to −0.1; *p* = 0.005). Aminophylline was also administered more frequently in infants requiring more respiratory support (OR 15.53, 95% CI 10.28 to 23.48; *p* < 0.0001).

Neonatal diagnoses are reported in Table 2. Receiving aminophylline was more frequent in neonates with respiratory distress syndrome (OR 7.39, 95% CI 5.16 to 10.60; *p* < 0.0001), asphyxia (OR 1.64, 95% CI 1.16 to 2.33; *p* = 0.007), or prematurity (OR 16.09, 95% CI 11.33 to 22.86; *p* < 0.0001). On the other hand, receiving aminophylline was less frequent in neonates with pneumonia/sepsis/meconium aspiration syndrome (OR 0.50, 95% CI 0.36 to 0.71; *p* = 0.0001), congenital malformations (OR 0.15, 95% CI 0.01 to 0.86; *p* = 0.04), feeding problems (OR 0.00, 95% CI 0.00 to 0.71; *p* = 0.01), or hyperbilirubinemia (OR 0.22, 95% CI 0.11 to 0.40; *p* < 0.0001).

Discharge information is displayed in Table 3. Hospital length of stay was longer in neonates who received aminophylline (median difference 3 days, 95% CI 2 to 4 days; *p* < 0.0001). During hospital stay, more respiratory support (OR 11.41, 95% CI 8.13 to 16.00; *p* < 0.0001), intravenous line use (OR 21.57; 8.81 to 52.79; *p* < 0.0001), and antibiotic therapy (OR 10.49, 95% CI 6.12 to 19.33; *p* < 0.0001) were more frequently administered to neonates treated with aminophylline. Moreover, they also received prolonged continuous positive airway pressure (median difference 1 day, 95% CI 1 to 2 days; *p* < 0.0001). Notably, kangaroo mother care was more common in neonates who received aminophylline (OR 7.65, 95% CI 5.35 to 10.95; *p* < 0.0001), although they were less likely to receive exclusive breastfeeding (OR 0.44, 95% C0.31 to 0.62; *p* < 0.0001). In the end, the mortality rate was higher in neonates who received aminophylline (OR 13.90, 95% CI 9.18 to 21.17; *p* < 0.0001).

## 4. Discussion

Our study explored the use of methylxanthines in a large cohort of infants who were admitted to neonatal special care units in Sub-Saharan settings. Our findings offered an estimate of its prevalence in clinical practice and depicted the profile of the recipients in settings where information on this topic remains limited.

In our hospitals, methylxanthine treatment was performed using aminophylline due to the unavailability of caffeine, which is common in low-resource settings due to cost and shortage of the latter [11,12,16,17]. The World Health Organization recommends caffeine for the prevention and treatment of apnea in preterm infants, while other methylxanthines (such as aminophylline or theophylline) may be considered when caffeine is unavailable [10]. Among the advantages of caffeine, the ready-to-use oral formulation without mixing and the once-per-day intake reduce the workload for healthcare staff, whereas aminophylline is given multiple times a day and needs to be diluted before use [10].

Our data showed that around 12% of infants were treated with aminophylline in the special care units. Unfortunately, we could not compare such a figure with the actual prevalence of neonates eligible for methylxanthine treatment or with previous literature on this topic. As expected, aminophylline treatment was progressively more frequent in smaller infants, who are more likely to develop apnea of prematurity [5,18]. Nonetheless, we believe that very-low-birth-weight infants might have been undertreated, because one out of three infants did not receive aminophylline prophylaxis [11]. The actual reason for this is unknown, but it may be associated with drug shortages and/or local protocols.

Overall, neonates with prematurity-associated conditions (such as preeclampsia/eclampsia, twins, lower Apgar score, lower temperature and oxygen saturation, and need for respiratory support) had a reasonably higher chance of receiving aminophylline [5]. In addition, aminophylline was also more common in asphyxiated neonates and those born through assisted breech, who are likely to develop postnatal respiratory impairment. Despite the advantage of methylxanthines in such cases, the reasons for this impairment are still unclear [19,20]. On the other hand, aminophylline was less frequently given to neonates with a primary diagnosis not associated with apnea episodes (such as meconium-stained amniotic fluid, congenital malformations, feeding problems, or hyperbilirubinemia), according to international recommendations [10].

As predicted, discharge information displayed a picture of neonates treated with aminophylline who were sicker compared to the others. In fact, they required more therapeutic support (such as non-invasive respiratory support, intravenous lines, and antibiotic therapy) during a prolonged hospital stay, which is expected to be associated with the preterm condition [4]. This consideration about severity aligned with the increased mortality rate among this group of patients. Improving the survival of small vulnerable newborns in low-resource settings requires the optimization of available assets and additional human and technological resources [21]. In addition, sicker babies may benefit from a short-term follow-up visit after discharge, as suggested by a recent study on neonatal readmission [22].

Kangaroo mother care is strongly recommended in all preterm or low-birth-weight infants to improve neonatal outcomes such as mortality, infection, weight gain, and breastfeeding [11]. In our study, kangaroo mother care was more common in neonates who received aminophylline as appropriate according to the premature condition. However, there was still room for improvement in routine care, since many preterm infants did not receive kangaroo mother care.

This study has some limitations that should be considered by the reader. First, the retrospective design limited the availability of some information such as the dose and timing of aminophylline treatment; hence, we could not exclude that some suboptimal dose/timing of the therapy might have influenced the clinical outcomes. The reasons for protocol incompliance regarding aminophylline treatment could include aspects such as drug shortage or healthcare staff’s motivations [16], but this information was not available and could not be investigated. In addition, caffeine has been associated with fewer side effects compared to aminophylline [12] but, unfortunately, data on aminophylline-related side effects were not available in the registry records. Therefore, we could not draw any conclusions about adverse events after aminophylline treatment or make any comparisons with caffeine treatment. Second, the information retrieved from the clinical charts did not allow for a full evaluation of disease severity, which could have influenced treatment efficacy and clinical outcomes. Third, the generalizability of the findings should be limited to similar settings.

Our findings suggest that the current data registry may be improved by adding specific items about disease severity, dose/timing, and adverse events of methylxanthine treatment. Further studies may evaluate protocol deviations and their reasons in order to improve the quality of the treatment. Future development may locally implement caffeine use for apnea of prematurity and assess the improvement with a before–after study.

## 5. Conclusions

In two Sub-Saharan settings, methylxanthine treatment involved around 12% of infants admitted to special care units and was limited to aminophylline due to the unavailability of caffeine. Overall, the treatment was appropriately given to most eligible neonates, although a considerable subgroup of very preterm infants did not receive aminophylline prophylaxis and require further attention. Local healthcare providers may investigate protocol deviations and reasons to improve the quality of the treatment. Local registries may be updated by including more information on methylxanthine treatment such as dose, timing, and adverse events. Further studies may investigate the reasons for protocol incompliance regarding aminophylline treatment and healthcare staff’s opinions on such an aspect.

## Figures and Tables

**Table 1:** Baseline characteristics in neonates who did and did not receive aminophylline.

Variable	Neonates Who Received Aminophylline (*n* = 196)	Neonates Who Did Not Receive Aminophylline (*n* = 1478)	*p*-Value
Hospital:			0.32
Tosamaganga	98 (12.6)	679 (87.4)	
Wolisso	98 (10.9)	799 (89.1)	
Source of admission: ^a^			<0.0001
Homebirth	20 (7.4)	252 (92.6)	
Inborn	141 (11.3)	1102 (88.7)	
Outborn	33 (21.4)	121 (78.6)	
Preeclampsia/eclampsia:			<0.0001
Yes	9 (29.0)	22 (71.0)	
No	131 (10.1)	1169 (89.9)	
Not reported	56 (16.3)	287 (83.7)	
Primiparous:			0.49
Yes	47 (10.3)	410 (89.7)	
No	88 (11.9)	651 (88.1)	
Not reported	61 (12.8)	417 (7.2)	
Prolonged rupture of membranes:			0.63
Yes	32 (13.1)	213 (86.9)	
No	151 (11.3)	1182 (88.7)	
Not reported	13 (13.5)	83 (86.5)	
Amniotic fluid:			0.002
Clear	133 (11.8)	991 (88.2)	
Meconium-stained	20 (6.9)	270 (93.1)	
Not reported	43 (16.5)	217 (83.5)	
Mode of delivery: ^b^			<0.0001
Assisted breech	12 (33.3)	24 (66.7)	
Cesarean section	48 (9.0)	487 (91.0)	
Vaginal delivery	131 (12.9)	882 (87.1)	
Vacuum	1 (1.7)	59 (98.3)	
Single/multiple births:			<0.0001
Single births	134 (9.4)	1284 (90.6)	
Multiple births	52 (31.5)	113 (68.5)	
Not reported	10 (11.0)	81 (89.0)	
Delayed cord clamping:			0.09
Yes	41 (9.2)	406 (90.8)	
No	54 (11.4)	419 (88.6)	
Not reported	101 (13.4)	653 (86.6)	
Sex:			0.28
Females	88 (12.8)	600 (87.2)	
Males	108 (11.0)	878 (89.0)	
Gestational age:			<0.0001
<28 weeks	15 (93.7)	1 (6.3)	
28–32 weeks	30 (75.0)	10 (25.0)	
32–34 weeks	50 (43.9)	64 (56.1)	
35–36 weeks	44 (20.5)	171 (79.5)	
37–42 weeks	35 (3.2)	1048 (96.8)	
>42 weeks	0 (0.0)	35 (100.0)	
Not reported	22 (12.9)	149 (87.1)	
Birth weight classification: ^c^			<0.0001
Extremely low birth weight (<1000 g)	17 (89.5)	2 (10.5)	
Very low birth weight (1000–1499 g)	60 (64.5)	33 (35.5)	
Low birth weight (1500–2499 g)	94 (21.3)	347 (78.7)	
Normal birth weight (≥2500 g)	24 (2.2)	1081 (97.8)	
Apgar score at 5 min:			<0.0001
<7	56 (19.2)	236 (80.8)	
≥7	95 (9.0)	955 (91.0)	
Not reported	45 (13.6)	287 (86.4)	
Temperature, °C ^c^	35.0 (34.2–35.8)	36.0 (35.2–36.7)	<0.0001
Transcutaneous oxygen saturation, % ^d^	94 (85–96)	95 (91–97)	0.005
Respiratory support: ^c^			<0.0001
Continuous positive airway pressure	92 (40.7)	134 (52.3)	
Oxygen therapy	63 (12.9)	424 (87.1)	
No support	40 (4.2)	905 (95.8)	

Data were summarized as *n* (%) or median (IQR). The percentages are row percentages. Data were not available for ^a^ 5, ^b^ 30, ^c^ 16, and ^d^ 174 subjects.

**Table 2:** Neonatal diagnoses in neonates who did and did not receive aminophylline.

Diagnosis	Neonates Who Received Aminophylline (*n* = 196)	Neonates Who Did Not Receive Aminophylline (*n* = 1478)	*p*-Value
Pneumonia/sepsis/meconium aspiration syndrome:			0.0001
Yes	47 (7.6)	569 (92.4)	
No	149 (14.1)	909 (85.9)	
Congenital malformations:			0.04
Yes	1 (2.0)	50 (98.0)	
No	195 (12.0)	1428 (88.0)	
Respiratory distress syndrome:			<0.0001
Yes	67 (40.8)	97 (59.1)	
No	129 (8.5)	1381 (91.5)	
Feeding problems:			0.01
Yes	0 (0.0)	40 (100.0)	
No	196 (12.0)	1438 (88.0)	
Hyperbilirubinemia:			<0.0001
Yes	13 (3.5)	357 (96.5)	
No	183 (14.0)	1121 (86.0)	
Asphyxia:			0.007
Yes	49 (16.4)	249 (83.6)	
No	147 (10.7)	1229 (89.3)	
Prematurity:			<0.0001
Yes	146 (39.1)	227 (60.9)	
No	50 (3.8)	1251 (96.2)	
Wet lung:			0.19
Yes	11 (7.9)	128 (92.1)	
No	185 (12.0)	1350 (88.0)	

Data were summarized as *n* (%) or median (IQR). The percentages are row percentages.

**Table 3:** Discharge information of neonates who did and did not receive aminophylline.

Discharge Information	Neonates Who Received Aminophylline (*n* = 196)	Neonates Who Did Not Receive Aminophylline (*n* = 1478)	*p*-Value
Hospital length of stay, days	8 (3–19)	5 (2–7)	<0.0001
Max respiratory support during hospital stay:			<0.0001
Continuous positive airway pressure	108 (55.1)	148 (10.0)	
Oxygen therapy	38 (19.4)	342 (23.2)	
No support	39 (19.9)	862 (58.3)	
Not reported	11 (5.6)	126 (8.5)	
Duration of the continuous positive airway pressure, days	3 (2–5)	2 (1–3)	<0.0001
Intravenous line:			<0.0001
Yes	185 (94.4)	873 (59.1)	
No	5 (2.5)	509 (34.4)	
Not reported	6 (3.1)	96 (6.5)	
Antibiotic therapy	181 (92.3)	790 (53.5)	<0.0001
Kangaroo mother care ^a^	79 (41.8)	123 (8.6)	<0.0001
Feeding method:			<0.0001
Maternal milk	132 (67.3)	1220 (82.5)	
Mixed	27 (13.8)	156 (10.5)	
Formula	6 (3.1)	51 (3.5)	
Not reported	31 (15.8)	51 (3.5)	
Discharge status: ^b^			<0.0001
Alive	116 (59.2)	1364 (93.0)	
Deceased	69 (35.2)	55 (3.7)	
Transferred	11 (5.6)	48 (3.3)	

Data were summarized as *n* (%) or median (IQR). The percentages are column percentages. Data were not available in ^a^ 48 and ^b^ 11 subjects.

## Data Availability

Data are available upon motivated request to the corresponding author. The data are not publicly available due to data set has not been published and the data has been retrieved from the international registry that has limited access.
